# A High-Throughput Enzyme Assay for Organophosphate Residues in Milk

**DOI:** 10.3390/s101211274

**Published:** 2010-12-09

**Authors:** Rupesh K. Mishra, Kanchanmala Deshpande, Sunil Bhand

**Affiliations:** Biosensor Laboratory, Department of Chemistry, BITS, Pilani-K K Birla Goa Campus, Goa, 403726, India; E-Mails: rupeshm@bits-goa.ac.in (R.K.M.); kanchan@bits-goa.ac.in (K.D.)

**Keywords:** stabilized butyrylcholinesterase, organophosphate, milk, high throughput, chemiluminescence, bioassay

## Abstract

A rapid, high-sensitivity, chemiluminescence (CL) enzyme assay for the determination of organophosphate (OP) residues in milk is presented. The assay for quantification of OP residues in milk is based on the inhibition of enzyme butyrylcholinesterase (BuChE). BuChE was stabilized and preloaded in 384 well plates at 30 °C. The assay permits rapid determination of OPs in milk within 12 min including an incubation step. The enzyme assay was tested for individual and mixtures of OPs such as methyl paraoxon (MPOx), methyl parathion (MP) and malathion (MT) in milk to evaluate their synergistic effect on BuChE inhibition. Good linearity was obtained in the range 0.005–50 μg·L^−1^ for MPOx and 0.5–1,000 μg·L^−1^ for MP as well as MT in milk. Mean recovery of 93.2%–98.6% was obtained for MPOx spiked milk samples with 0.99%–1.67% reproducibility (RSD). The proposed method facilitated rapid screening of milk samples in 384 well plate formats with further miniaturization presented in 1,536 well plates.

## 1. Introduction

Contamination of milk by toxic substances causes a serious problem to the agricultural and dairy industry. Among the various milk contaminants, organophosphate compounds (OPs) are among the most important due to their high toxicity, even at very low residual concentrations. Although, OPs have relatively low persistence in the environment, there are a number of ways in which they can reach milk [1]. The presence of OP residues in milk has already been reported by many researchers [2–6], and is mainly due to the ability of OPs to covalently link with milk proteins [7]. Reported high exposure of OPs and its concurrent toxicological effects in developing countries, like India and China is a serious concern [8,9]. There is a need for fast screening techniques, especially for neurotoxic insecticides in food items that are consumed to a great extent by vulnerable groups such as young children and infants. The European Union has set a very low limit for pesticides in baby food. According to this regulation, infant formulae must not contain residues of individual pesticides at level exceeding 10 μg kg^−1^, which is in practice the minimum detectable level using the officially accepted detection methods [10].

Reported high levels of residual OPs in human body and their high toxicity at trace levels has forced many scientists to develop highly sensitive, selective, rapid and reliable analytical method for the determination of OPs. Apart from the standard analytical method used for OP analysis, biosensors have emerged as a potentially sensitive technique with the added advantage of toxicity assessment. Enzyme inhibition-based biosensors using various enzymes have been reported for OP analysis [11–15] but their application to real samples such as milk remains a challenge, which is also evident from the scarcity of reported literature. Biosensors based on cholinesterase enzymes (ChEs), have emerged as a sensitive and selective technique for toxicity assessment in food and agricultural applications [16]. The inhibition of acetylcholinesterase (AChE) by OP in milk reported by Beam and Hankinson [17] is considered as one of the pioneer works in this field. Sensitive determination of methyl paraoxon (MPOx) using AChE has been reported [18]. OP analysis using butyrylcholinesterase (BuChE) inhibition has been reported using electrochemical biosensors [19–21]. Few reports are available for sensitive optical detection in high throughput assay formats. Measurement of OP-induced inhibition using BuChE provides comparatively higher stability and sensitivity towards pesticide analysis over AChE [22].

Reliability of an enzyme biosensor is dictated by the stability of the enzyme used, as a biosensor may often be stored for weeks or months prior to its use. Enzymes such as ChEs provide limited operational and storage stability at room temperature. It has been reported that unfolding of proteins can be prevented by using stabilizers that remain in the amorphous phase with the protein and hydrogen bond to the protein in place of water during drying [23,24]. In the present work, BuChE stabilization has been achieved at room temperature with extended shelf life.

Development of an OP biosensor for use as a screening assay for milk samples is of paramount interest. For high-throughput analysis, enzyme inhibition-based biosensors coupled with chemiluminescence (CL) technique in 384 and 1,536 well plate formats, has attracted immense interest over the past two decades. Co-exposure to OPs such as MPOx, methyl parathion (MP) and malathion (MT) is very common in the environment. Thus, toxicity study of these OPs in combination is of imperative significance [25]. Although the effects of individual OPs on ChEs activity have been studied for decades, the neurotoxicity of mixtures is still poorly understood.

Herein, we present a rapid miniaturized assay in 384 and 1,536 well plate format for OP residues in milk. The assay utilizes BuChE inhibition with CL technique, for the determination of highly toxic OPs such as MPOx, MP and MT in milk. The synergistic effect of OPs mixture on BuChE inhibition in milk sample was also studied. A novel stabilization protocol was utilized in the present study with preloaded BuChE in micro well plates. The enzyme showed significant stability over a period of six weeks.

## 2. Experimental Section

### 2.1. Chemicals and Instruments

Butyrylcholinesterase (E.C.3.1.1.8) from *Equine serum,* choline oxidase (ChOx) (E.C.1.1.3.17) from *Alkaligenes species,* peroxidase (HRP) (E.C.1.11.1.7) from *Horseradish,* butyrylcholine chloride, choline chloride, trehalose, 5-amino-2,3-dihydro-1,4-phthalazinedione (Luminol) and protein standard, micro standard solution were purchased from Sigma Chemical Co. (St. Louis, MO, USA)*,* Methyl paraoxon PESTANAL™ grade purity 96.3 area·%, methyl parathion PESTANAL™ grade purity 99.9 area·% and malathion PESTANAL™ grade purity 97.3 area·% were purchased from Riedel-de Haën (Germany). Hydrogen peroxide (30%), acetonitrile, sodium phosphate dibasic, sodium phosphate monobasic and other chemicals were of GR grade, Merck (Germany). Dextrose, anhydrous, A. R. was obtained from High Media Laboratories (Mumbai, India). Multi label Reader Victor™ X4 offers a high sensitivity luminescence measurement system with capability to measure both 384 and 1,536 well plate format. The detector used in the system is a red photomultiplier tube, capable of low photon counting. Microtiter plates Optiplate 384 (Nunc, Denmark), 1,536-Well Plates (Corning, USA) and micro pipettes (Eppendorf, Germany) were used for the assays. All other reagents used were of GR grade.

### 2.2. Reagent Preparation

Phosphate buffer (PB) 0.1 M, pH 7.4 was prepared by mixing sodium dihydrogen phosphate monohydrate GR (0.1 M, pH 4.4) and di-sodium hydrogen phosphate anhydrous GR (0.1 M pH 9.2) using ultra pure water. Stock solutions (1 mg·mL^−1^) of MPOx, MP and MT were prepared in 5% acetonitrile. Stock solutions of BuChCl (0.1 M), BuChE (160 U/mL), ChOx (8 U/mL) and HRP (1 U/mL) were prepared in PB and stored at 4 °C. Working solutions were prepared every day by appropriate serial dilutions in 0.1 M PB. Luminol solution was prepared by dissolving 4 mg of luminol in 2 mL 0.1 M, NaOH and making up the volume to 20 mL by 0.1 M PB, pH 7.4.

### 2.3. Bio-Assay Principle

**Scheme 1.**
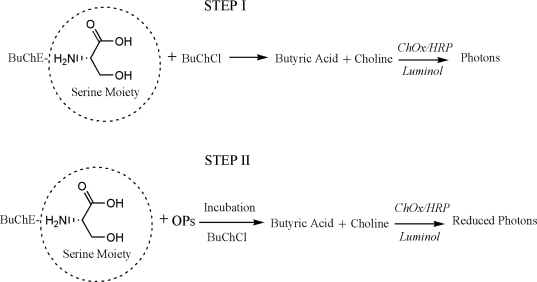
Principle of chemiluminescence based enzyme inhibition assay for OPs.

The presented assay is based on the inhibition of BuChE by OP residues. During the inhibition, serine hydroxyl moiety in the BuChE active site is phosphorylated. The serine hydroxyl group, blocked by a phosphoryl moiety is no longer able to participate in the hydrolysis of BuChCl [26–28]. Assay principle is presented as Scheme 1. The percentage of inhibited enzyme activity (I%) that results after exposure to the inhibitor is quantitatively related to the inhibitor concentration according to following equation [14]:

(1) $$\text{I}\%=({\text{A}}_{0}-{\text{A}}_{\text{i}}/{\text{A}}_{0})\times 100$$

where A_0_ is the activity in the absence of inhibitor and A_i_ is the activity in the presence of inhibitor. CL technique was exploited to determine the activity of stabilized BuChE.

### 2.4. Stabilization of BuChE and Assay Development

For enzyme stabilization, dextrose and trehalose were dissolved in protein standard solution (2% w/v). Different compositions of stabilizer and BuChE (0.5:1, 1:1, 1:2) were prepared. We stabilized BuChE with dextrose in protein standard solution (1 mL/amp: 1 mg BSA/mL in 0.15 M NaCl, 0.05% NaN_3_). The assay protocol for inhibition studies is as follows: The stabilized BuChE (0.5 μL of 0.08 U) was dispensed in the 384 microwell plate and were dried at room temperature. Stabilized BuChE forms thin film like layer at the bottom of the well. Subsequently, 5 μL of inhibitor (in PB or Milk) was added to well and incubated for 10 min. The reaction was followed by addition of reaction mixture (14.5 μL) consisting of BuChCl (0.5 mM), ChOx (0.004 U), HRP (0.0008 U) and luminol (1 mM). The number of photons emitted was recorded. A washing step was also performed after incubating stabilized BuChE with OPs.

### 2.5. Analysis in Milk

Commercial milk samples containing 0.5% fat were purchased from a local market in Goa, India. Milk samples were filtered using only a 0.2 μm filter (Whatman USA) and diluted prior to analysis. Matrix matching studies were carried out by preparing different dilutions of milk in PB (1:10, 1:100, 1:500, 1:1,000, and 1:2,000). Milk samples were spiked with individual pesticides (MPOx, MP&MT) and their mixture taking MPOx as a reference. The concentration of OPs in mixture is as follows. *Mixture 1*: MPOx, MP and MT (1 μg·L^−1^ each), *Mixture 2*: MPOx (1 μg·L^−1^) and MP, MT (10 μg·L^−1^ each), *Mixture 3*: MPOx (1 μg·L^−1^) MP and MT (100 μg·L^−1^ each). For each inhibition assay, 5 μL of milk sample spiked with OPs mixture was used and assay was carried out as described in assay protocol.

## 3. Results and Discussion

### 3.1. Stabilization of BuChE

The enzyme BuChE is sensitive to temperature fluctuations. Different stabilizer compositions reported in the literature are presented in Table 1. While AChE stabilization has been reported, room temperature stability of BuChE for bioassays has not been reported. We have investigated the stabilization of BuChE using dextrose and trehalose in protein standard solution. The other two enzymes namely ChOx and HRP are added in the presented assay after the inhibition step. Thus, their storage stability at 4 °C was also evaluated using the developed assay. Intraday and inter-day stability of ChOx and HRP was also studied up to six weeks (provided as Supplementary Material).

**Figure 1.**
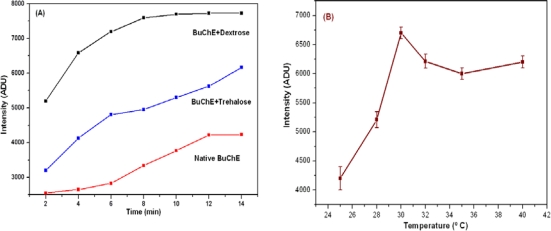
The Figure marked by **(A)** is CL intensity profile for native BuChE and stabilized BuChE with trehalose and dextrose in protein standard solution; the Figure marked by **(B)** shows Effect of temperature on the CL intensity of the stabilized BuChE in micro well plate with 0.5 mM BuChCl, 0.1 M PB, pH 7.4.

The kinetic profile of native and stabilized BuChE at 30 °C is presented in Figure 1(A). The intensity profile shows increasing trend where intensity saturates after 6 min for dextrose stabilized BuChE. This stability in the intensity also decreases the analysis time. It is clear that with stabilization, we could achieve almost double enhancement in the intensity than native enzyme. Among the two stabilizers studied, dextrose in protein standard solution provides higher stability over trehalose. With tested stabilizer composition, the data obtained is highly reproducible for longer duration. The effect of temperature on stabilized BuChE activity was studied using dextrose solution in the range 25–40 °C. Results are presented in Figure 1(B). It is evident from the observed results that the activity of stabilized BuChE was found much stable in the range 30–40 °C.

### 3.2. Enzyme and Substrate Optimization

**Figure 2.**
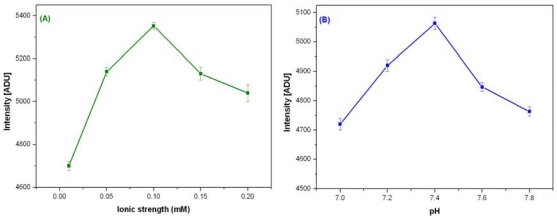
The Figure **(A)** variation of CL intensity profile for stabilized BuChE at different ionic strength of buffer and Figure **(B)** Effect of pH on the activity of stabilized BuChE in micro well plate with 0.5 mM BuChCl, 0.1 M PB, pH 7.4.

The activity of BuChE in tri-enzyme system also depends on the buffer, pH and ionic strength. The activity of stabilized BuChE was optimized by varying ionic strength (0.01–0.2 M) and pH (7–7.8) for PB. BuChE shows maximum activity at 30 °C with 0.1 M PB, pH 7.4. Figure 2(A,B) shows optimization of ionic strength and pH for BuChE reaction.

Experiments were performed initially with varied concentrations of BuChE (0.04–0.32 U) and subsequently with BuChCl (0.03–3 mM) under optimized experimental conditions. The Michaelis constant, K*_M_* apparent was calculated 0.27 mM for BuChE using Lineweaver-Burk plot.

### 3.3. Assay Performance in PB

**Figure 3.**
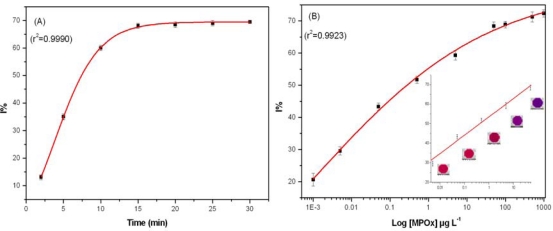
**(A)** Inhibition profile obtained for 50 μg·L^−1^ MPOx at different incubation time using 0.5 mM BuChCl at 30 °C; **(B)** Calibration curve obtained for MPOx in PB using 0.08 U BuChE with 0.5 mM BuChCl, 0.004 U ChOx, 0.0008 U HRP, 0.1 M PB, pH 7.4 at 30 °C. (*Inset: linear range for MPOx with CL image*).

Assay performance was tested with MPOx as a model inhibitor in PB with 0.5 mM BuChCl as a compromise between enzyme unit and substrate concentration used to avoid substrate deficiency. In the studied range of BuChCl, substrate induced inhibition was not observed. Incubation time is a key parameter in pesticide residue analysis. Thus, effect of different incubation time (2, 5, 10, 15, 20, 25, and 30 min) on BuChE activity was studied with 50 μg·L^−1^ MPOx. A sigmoid curve was obtained when data for different incubation time was plotted against I% and results are presented as Figure 3(A).

A good linearity was observed up to 10 min, where BuChE showed 60% inhibition. It is important to note that after 10 min incubation, no significant increase in I% was observed. Therefore all the measurements were performed with 10 min incubation. The efficiency of proposed assay was tested by exposing BuChE to different MPOx concentration in PB. A calibration curve was constructed for MPOx in PB (as a reference) prior to milk analysis. Figure 3(B) shows the percentage of BuChE inhibition caused by different MPOx concentration. No significant difference in the degree of inhibition was observed when an additional washing step was introduced after incubation with pesticide. Having only BuChE as an immobilized enzyme component facilitates specific inhibition of BuChE by OPs, which in other case having all three enzymes (BuChE/ChOx/HRP) together in the well was not observed.

Each experimental data point is mean of three inhibition assays whereas in each assay, triplicate measurements were performed for each MPOx concentration. A good linearity was found in the range 0.005–50 μg·L^−1^ with equation of line: Y = 53.4754 + 9.3608·X, r^2^ = 0.9931. The BuChE assay could achieve a lower limit of detection up to 0.001 μg·L^−1^ for MPOx in 384 well plate formats, which is much lower than reported values in the literature [11,15,18,27].

### 3.4. Assay Performance in Milk for OPs Determination

**Figure 4.**
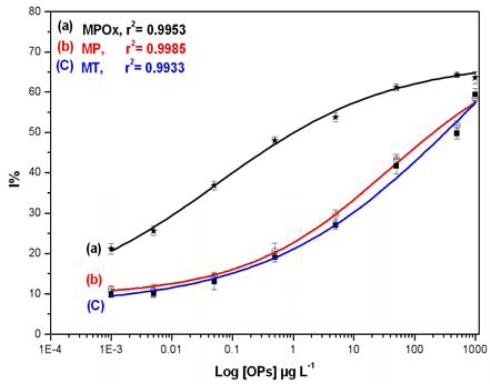
Inhibition profile obtained for (a) MPOx (b) MP and (c) MT spiked milk samples using 0.08 U BuChE. The assays were carried out in 384 well plate formats with 10 min incubation time.

Inhibition studies were extended to analyze three OPs, namely MPOx, MP and MT, in milk samples. Different concentrations of individual OPs (0.001–1,000 μg·L^−1^) in milk were mixed with BuChE and allowed to incubate for 10 min in dark. The data obtained was used to construct standard inhibition curve relating to OPs concentration in milk and presented in Figure 4.

As indicated in Figure 4, the MPOx curve (a) shows maximum inhibition up to 65%, whereas MP (b) and MT (C) curve shows maximum inhibition up to 60%. MPOx shows 15–20% more inhibition than other two OPs in the lower concentration range. Linear range, Limit of quantification (LOQ) and Inhibitory concentration (IC) values were calculated and presented in Table 2. Lowest concentration in the linear range is represented as LOQ whereas, concentration corresponding to 50% inhibition is represented as IC_50_. The higher toxicity of MPOx as against MP and MT is clearly evident from the significant difference in their IC_50_ values and linear range. This investigation also revealed the sensitivity of BuChE towards MPOx as against other two OPs.

### 3.5. Analysis of MPOx in real Milk Samples

The developed assay has been applied to the analysis of real milk samples to demonstrate the possible presence of MPOx residues. Real milk samples were tested using the bioassay with and without spiking MPOx standard solution. Milk samples were spiked with different MPOx concentration so that the final concentration lied within calibration range. Results obtained are presented in Table 3. In spiked milk samples, recoveries were found in the range 93.2–98.6% with % RSD 0.99–1.67. Measurements were performed in triplicates and each experiment was carried out three times. The obtained % RSD values support the reproducibility of developed assay in milk. Lower recoveries up to 93.2% in the studied samples denote absence of MPOx in the sample or presence below the detection limit of the developed assay.

### 3.6. Reproducibility and Specificity

Reliability and reproducibility of BuChE inhibition assay was studied over an extended period of six weeks. Inhibition assays were performed for interbatch and intrabatch studies. Measurements were performed daily. The % RSD for successive MPOx inter-batch assay (N = 15) was 2.5. For intra batch assay over the period of six weeks, the % RSD is calculated as 3.5 (N = 40).

### 3.7. Analysis of Mixture of OPs

**Figure 5.**
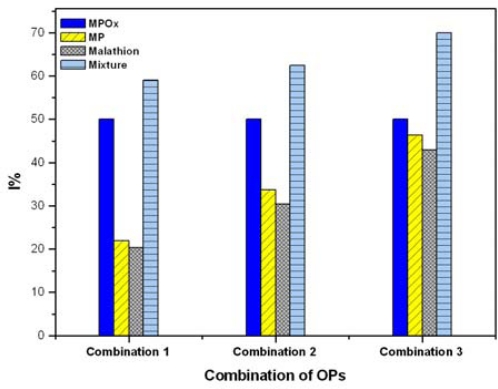
Inhibition pattern obtained for tertiary pesticide mixtures using stabilized BuChE. *Mixture combination 1*: MPOx, MP and MT (1 μg·L^−1^ each), *combination 2*: MPOx (1 μg·L^−1^) and MP, MT (10 μg·L^−1^ each), *combination 3*: MPOx (1 μg·L^−1^) MP and MT (100 μg·L^−1^ each).

The interactive effects (additive, synergistic, and antagonistic) of mixture of OPs were investigated using MPOx as a reference analyte. Milk samples spiked with mixtures of MPOx, MP and MT were studied for inhibition of BuChE. Figure 5 shows interactive effects of MPOx, MP and MT in three pesticide mixture combinations. For each combination, four bars are presented. The first three bars represent I% due to individual analyte and the fourth bar represent interactive effect of mixture. The objective of our study was to determine effect of MP and MT (over the varied concentration range) on fixed MPOx concentration. Throughout the inhibition curve, we observed that total inhibition induced by OPs mixture was not simply additive but synergistic (lower than the sum of individual inhibition values). From I% of the mixture, it is clear that inhibition by mixture is dominated by most potent inhibitor (*i.e.*, MPOx). The obtained results also match with the other reports [30,31].

### 3.8. Assay Miniaturization in 1,536 Well Plate

Miniaturization of assays enables the handling of low volume samples, reduction in reagent consumption, minimizes waste generation and increased sample throughput. The developed assay was further miniaturized from 384 to 1,536 well plate formats with a total assay volume 8.5 μL without compromising assay integrity. The following remarkable features were observed as result of miniaturization of assay: (i) reduction in incubation time by half (from 10 to 5 min) (ii) reduction in IC values by half (iii) two fold reduction in assay volume (iv) broad linear range (0.0005–0.5 μg·L^−1^) and (v) much higher sensitivity achieved up to 0.0005 μg·L^−1^. Comparative study of inhibition parameters in 384 and 1,536 well plate format is summarized in Table 4.

### 3.9. Long Term Stability of BuChE

**Figure 6.**
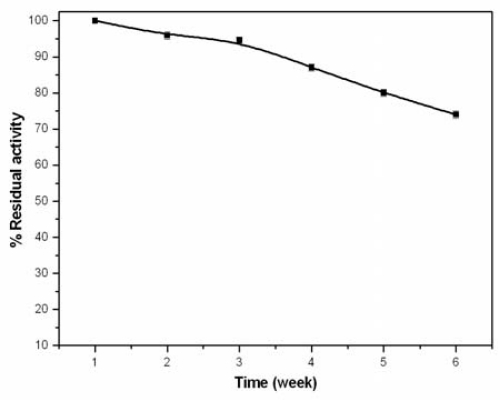
Room temperature activity profile of the stabilized BuChE enzyme.

The stabilized BuChE was preloaded in 1,536 well plates and dried at room temperature. Preloading was done to reduce handling time and asses storage stability at room temperature. The well plate was monitored periodically for activity measurement up to six weeks. Figure 6 shows the activity profile of stabilized BuChE, where each data point is the mean of three measurements. Stabilized BuChE retained more than 90% activity in the first two weeks whereas at the end of six weeks, BuChE retained 74% of activity. The extended stability of the enzyme is attributed to the added dextrose solution which facilitates stronger interaction of H-OH bond between protein and carbohydrate. This bond provides a higher conformational stability to BuChE. The reproducibility of the stabilized BuChE was studied and good %RSD *i.e.*, below 3.5% was obtained for the interday assay over six weeks.

## 4. Conclusions

The 384 well plate BuChE-based assay reported here enabled low level determination of individual OPs in milk sample with a limit of detection of 0.001 μg·L^−1^. Long term stability of enzymes in well plates for up to six weeks, short total analysis time (12 min) and high reproducibility are the key features of the presented work. The assay was successfully tested to study interactive effect of mixture of three common OP residues, MPOx, MP and MT, in milk. Recovery rates for MPOx in spiked milk samples lay between 93.2%–98.6%. The proposed method facilitates rapid analysis of milk samples in 384 well plate formats with further demonstrated miniaturization in 1,536 well plate formats. The detection limit was found out to be 0.0005 μg·L^−1^ of MPOx in milk in 1,536 well plate formats.

## Figures and Tables

**Table 1.** Various stabilizer compositions reported for Cholinesterase enzyme.

**S. No.**	**Enzyme**	**Stabilizer Composition**	**References**
1.	AChE	Gelatine and or/albumin containing trehalose as film.	US Patent 5624831 [29]
2.	AChE	Dextran-sulphate/sucrose mixture and polygalacturonic acid/sucrose mixtures.	Vakurov *et al*. 2005 [24]
3.	AChE	Mixture of glucose, trehalose and gelatine.	Weetall *et al.* 2004 [12]
4.	BuChE	Dextrose in protein standard solution (1 mL/amp: 1 mg BSA/mL in 0.15 M NaCl, 0.05% NaN_3_).	This work

**Table 2.** Analytical figures of merit for the developed bioassay in spiked milk.

**Analyte added**	**Linear range (μg·L^−1^)**	**LOQ (μg·L^−1^)**	**IC_50_ (μg·L^−1^)**
MPOx	0.005–50	0.001	1.02
MP	0.5–1,000	0.5	202.02
MT	0.5–1,000	0.5	326.70

**Table 3.** Analysis of MPOx in real milk samples and recovery studies.

**Samples**	**MPOx spiked μg·L^−1^**	**MPOx found μg·L^−1^**	**% Recovery (n = 3)**	**% RSD**
Milk-1	0.0	BDL	---	---
Milk-1	0.5	0.468	93.6 ± 0.93	0.99
Milk-1	5.0	4.820	96.4 ± 1.11	1.15
Milk-2	0.0	BDL	---	---
Milk-2	0.5	0.478	95.6 ± 1.03	1.07
Milk-2	5.0	4.860	97.2 ± 1.40	1.44
Milk-3	0.0	BDL	---	---
Milk-3	0.5	0.493	98.6 ± 1.0	1.01
Milk-3	5.0	4.660	93.2 ±1.56	1.67

* BDL: Below detection limit of developed bioassay *i.e.*, 0.001 μg·L^−1^.

**Table 4.** Analytical performance of the bioassay in 384 and 1,536 well plate format.

**Parameters**	**BuChE (Unit/Assay)**	**Incubation time (min)**	**IC_30_ (μg·L^−1^)**	**Total assay volume (μL)**
384-well assay	0.08	10	0.014	20
1,536-well assay	0.04	5	0.0053	8.5
